# Supplementary material for: Magnon interactions in a moderately correlated Mott insulator
Source: Nat Commun. 2024 Jun 24;15:5348. doi: 10.1038/s41467-024-49714-y (PMC11196644; doi:10.1038/s41467-024-49714-y)
Supplement: Supplementary file 1 — Supplementary Information [file 41467_2024_49714_MOESM1_ESM.pdf]

Supplementary Information for
Magnon Interactions in a Moderately Correlated Mott Insulator

Q. Wang *et al.*

Supplementary Note 1. Fitting of RIXS spectra

We analyze the low-energy part of the RIXS spectra by fitting globally across the two light polarisations to extract the single- and bi-magnon excitations. Elastic scattering is described by a Gaussian function with the width slightly larger than the instrumental resolution due to unresolved phonon modes. The single-magnon excitation is modeled by a damped harmonic oscillator. Taking into account the thermal population factor, the corresponding RIXS intensity from single-magnon excitation $S(\mathbf{Q}, \omega)$ is given by:

$$S(\mathbf{Q}, \omega) = \frac{\chi_0''}{2\omega_0(1 - e^{-\hbar\omega/k_\mathrm{B}T})} \left[\frac{\Gamma/2}{(\omega - \omega_0)^2 + (\Gamma/2)^2} - \frac{\Gamma/2}{(\omega + \omega_0)^2 + (\Gamma/2)^2} \right] \tag{1}$$

where $\hbar$ is the reduced Planck constant and k_B is the Boltzmann constant. We have convoluted $S(\mathbf{Q}, \omega)$ with the instrumental energy resolution function. Pole energy of the single-magnon is defined as $\omega_1 = \sqrt{\omega_0^2 - (\Gamma/2)^2}$. The bi-magnon component is described by a Gaussian function. A second-order polynomial is added to mimic the background. Fits of the RIXS spectra are displayed in Supplementary Fig. 1.

Supplementary Note 2. Magnon dispersions in $SrCuO_2$ and $CaCuO_2$

Previous RIXS studies reveal that single-magnon excitation in $CaCuO_2$ (CCO) also displays a large zone-boundary dispersion implying a moderate correlation strength similar to $SrCuO_2$ (SCO) [1, 2]. We thus applied our fitting model to the magnon dispersion in CCO reported in ref. [1]. As shown in Supplementary Fig. 2, global fitting to the experimental dispersion is satisfactory and yields $U/t = 6.8$, which is slightly larger than that of SCO. The complete fitting parameters are listed in Table I of the main text. The results reveal that there is an anti-correlation between the existence of a large and momentum-dependent quantum renormalization and the strength of electronic correlations as measured by U/t. In both compounds, quantum fluctuations are found to strongly influence the magnetic ground state and its excitations—see Figs. 3 and 4 of the main text.

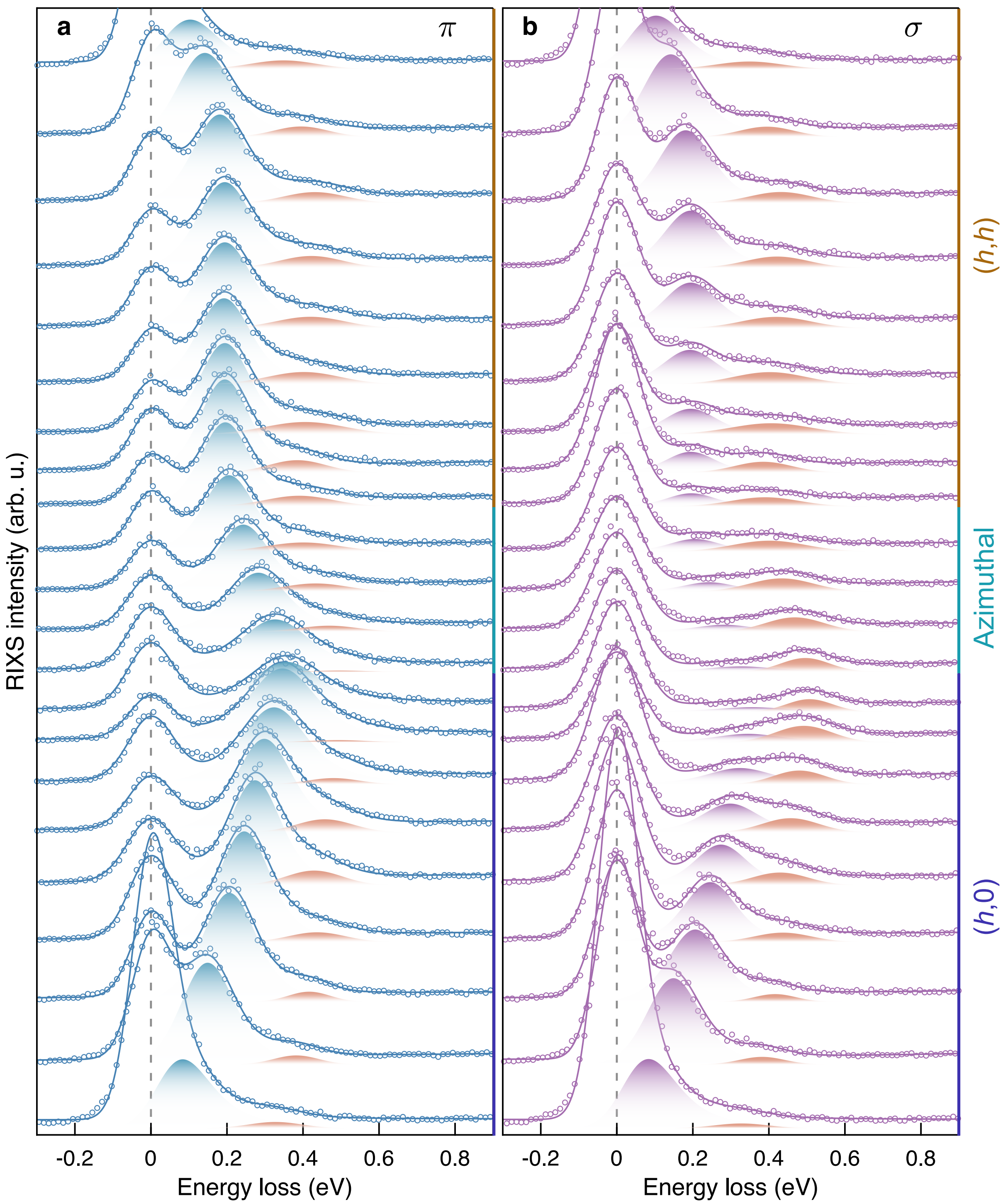

Supplementary Fig. 1. Fittings of RIXS spectra obtained with π (a) and σ (b) incident light polarisations. The solid lines are fits to the data. The grey dashed lines mark the zero energy loss, which is characterized by the fitted elastic peak position. Blue and purple shaded areas indicate the fitted components of single-magnon for π and σ polarisations, respectively. Orange shaded areas indicate the fitted components of the bi-magnon.

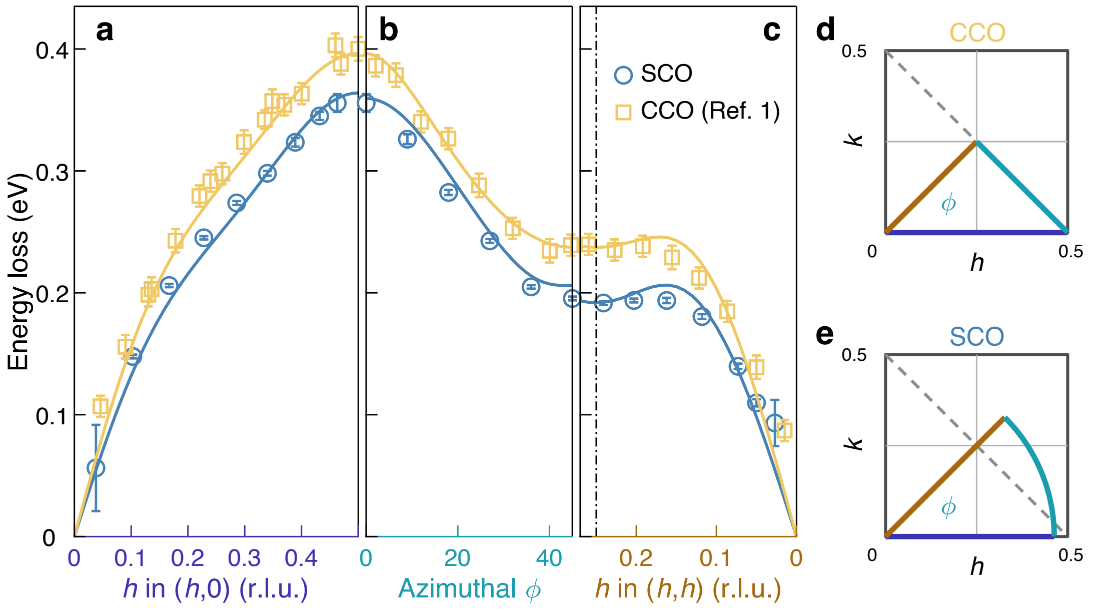

Supplementary Fig. 2. Magnon dispersions in $SrCuO_2$ and $CaCuO_2$. (a-c) display the magnon dispersions in SCO (blue open circles) and CCO (yellow open squares) and the respective fits (solid curves) using the Hubbard model as described in the main text. The momentum trajectories are shown in (d) and (e) for CCO and SCO, respectively. Error bars are determined from the fitting uncertainty. Data on CCO are adapted from ref. [1].

Supplementary Note 3. One-band Hubbard model with a constant quantum renormalization factor

A Heisenberg Hamiltonian derived from the one-band t-U Hubbard model has been commonly used to describe the magnon excitations in undoped cuprates [1, 3]:

$$\hat{\mathcal{H}} = J\sum_{\langle i,j\rangle}\mathbf{S}_i\cdot\mathbf{S}_j + J_2\sum_{\langle i,i'\rangle}\mathbf{S}_i\cdot\mathbf{S}_{i'} + J_3\sum_{\langle i,i''\rangle}\mathbf{S}_i\cdot\mathbf{S}_{i''} + J_\square\sum_{\langle i,j,k,l\rangle}\left[\left(\mathbf{S}_i\cdot\mathbf{S}_j\right)\left(\mathbf{S}_k\cdot\mathbf{S}_l\right) + \left(\mathbf{S}_i\cdot\mathbf{S}_l\right)\left(\mathbf{S}_k\cdot\mathbf{S}_j\right) - \left(\mathbf{S}_i\cdot\mathbf{S}_k\right)\left(\mathbf{S}_j\cdot\mathbf{S}_l\right)\right] \quad (2)$$

where J, J_2, and J_3 are the first-, second-, and third-nearest-neighbour exchange couplings, and $J_\square$ is the ring exchange interaction. A projection from one-band Hubbard model gives $J = \frac{4t^2}{U} - \frac{24t^4}{U^3}$, $J_\square = \frac{80t^4}{U^3}$, $J_2 = J_3 = \frac{J_\square}{20}$. A constant $Z_c = 1.18$ [4] obtained in the large U/t limit is used to account for the quantum correction of the magnon dispersion. In Supplementary Fig. 3, we compare the fitting results to the magnon dispersion in SCO using this model (blue dashed) and the t-t'-t''-U model with a momentum-dependent Z_c (blue solid). The significantly improved quality of the fits for the latter model demonstrates that evaluation of quantum renormalization effect from magnon-magnon interactions is required to describe the magnon dispersion in moderately correlated SCO. Note that the t-U model fitting yields an unrealistically small correlation strength with $U/t = 4.3$ and $U = 0.79$ eV.

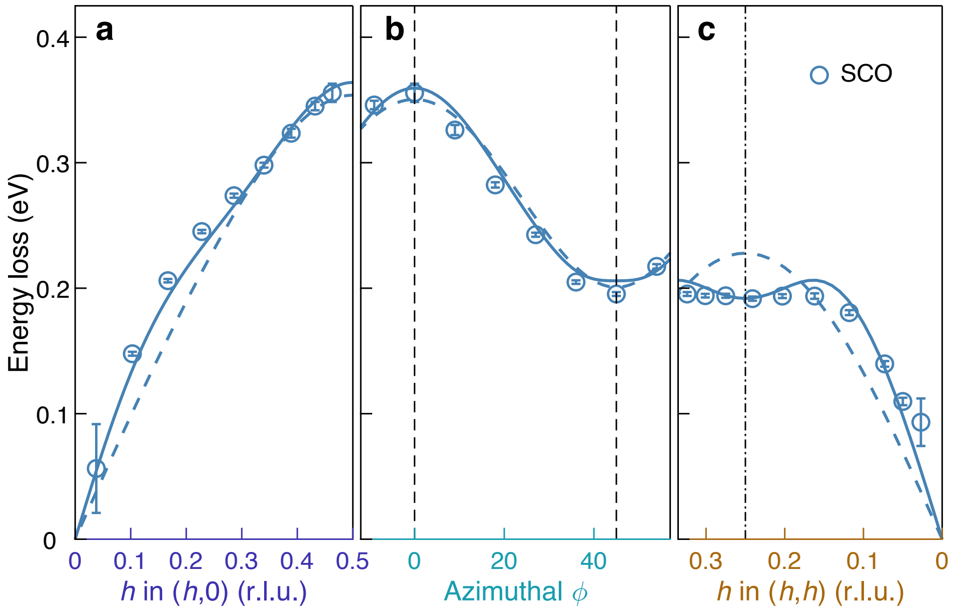

Supplementary Fig. 3. Comparison of fits to magnon dispersion in SCO using t-U Hubbard model with a constant Z_c (blue dashed) and the t-t'-t''-U model with a momentum-dependent Z_c evaluated from the magnon-magnon interactions (blue solid). Error bars are determined from the fitting uncertainty.

Supplementary Note 4. Spin structure factors from classical Monte Carlo simulations

Supplementary Fig. 4 displays representative spin structure factors from our classical Monte Carlo calculations. Antiferromagnetic Néel (columnar) order is found at small (large) t and t'/t region in the parameter space explored. Two types of incommensurate magnetic orders with wave vectors $\mathbf{Q}_{\mathrm{M1}} = (0.5 \pm \delta, 0.5)$ and $(0.5, 0.5 \pm \delta)$, or $\mathbf{Q}_{\mathrm{M2}} = (0.5 \pm \delta/\sqrt{2}, 0.5 \pm \delta/\sqrt{2})$ are found in between the antiferromagnetic Néel and columnar orders.

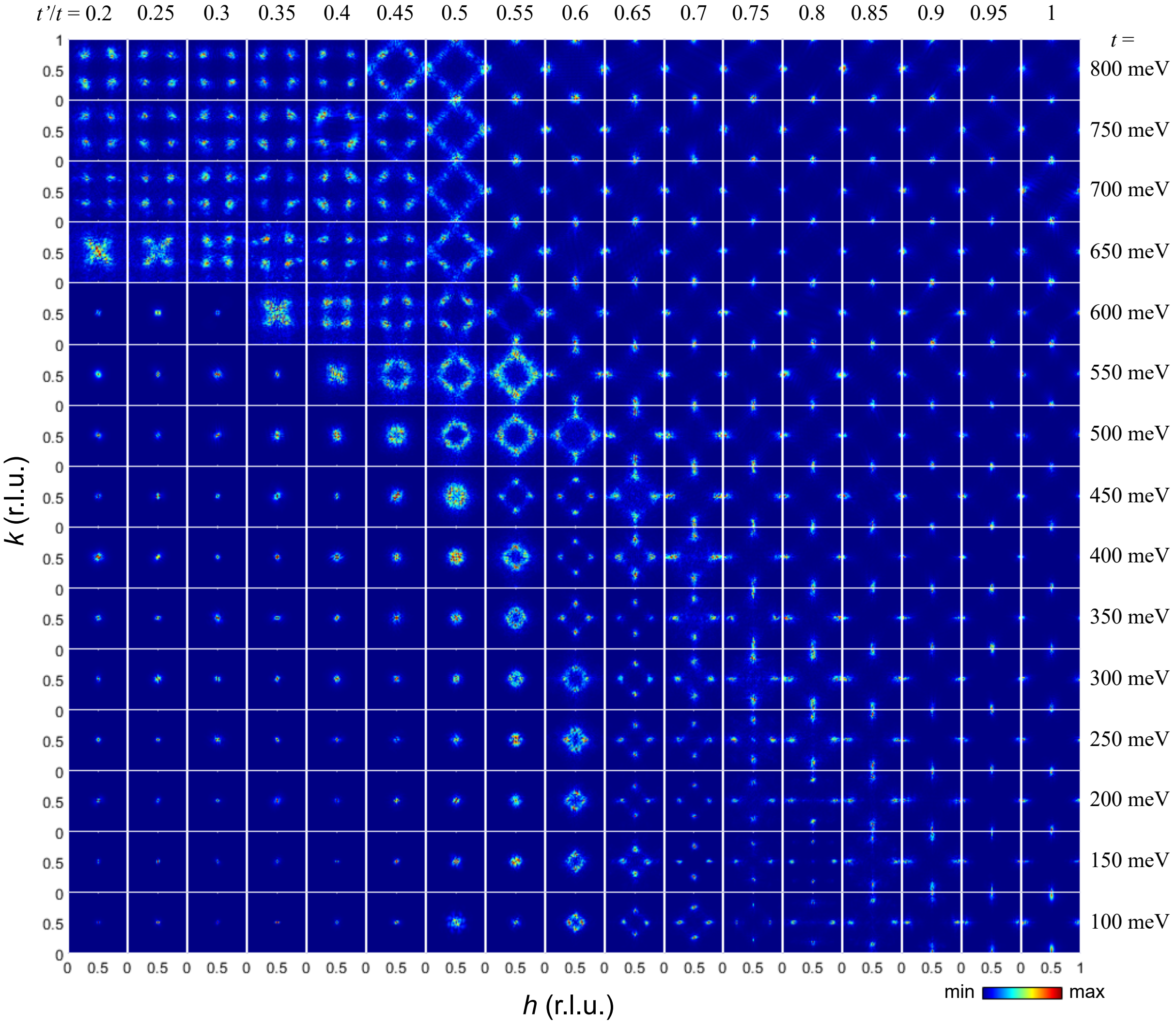

Supplementary Fig. 4. Representative spin structure factors from Monte Carlo calculations. Intensity distributions of the spin structure factors in the hk-plane are calculated as a function of t'/t and t.

Supplementary References

[1] Y. Y. Peng, G. Dellea, M. Minola, M. Conni, A. Amorese, D. Di Castro, G. M. De Luca, K. Kummer, M. Salluzzo, X. Sun, X. J. Zhou, G. Balestrino, M. Le Tacon, B. Keimer, L. Braicovich, N. B. Brookes, and G. Ghiringhelli, Nat. Phys. **13**, 1201 (2017).

[2] L. Martinelli, D. Betto, K. Kummer, R. Arpaia, L. Braicovich, D. Di Castro, N. B. Brookes, M. Moretti Sala, and G. Ghiringhelli, Phys. Rev. X **12**, 021041 (2022).

[3] R. Coldea, S. M. Hayden, G. Aeppli, T. G. Perring, C. D. Frost, T. E. Mason, S.-W. Cheong, and Z. Fisk, Phys. Rev. Lett. **86**, 5377 (2001).

[4] R. R. P. Singh, Phys. Rev. B **39**, 9760 (1989).
